# Sistema OLGA (*Operative Link on Gastritis Assessment*) como marcador para cáncer gástrico y displasia en una población colombiana de alto riesgo: estudio multicéntrico

**DOI:** 10.7705/biomedica.6995

**Published:** 2023-12-29

**Authors:** Yeison Harvey Carlosama, Claudia Patricia Acosta, Carlos Hernán Sierra, Carol Yovanna Rosero, Harold Jofre Bolaños

**Affiliations:** 1 Grupo de Biología Molecular de la Salud, Universidad Autónoma de Manizales, Manizales, Colombia Universidad Autonoma de Manizales Grupo de Biología Molecular de la Salud Universidad Autónoma de Manizales Manizales Colombia; 2 Grupo Interdisciplinario de Investigación en Salud y Enfermedad, Universidad Cooperativa de Colombia, Pasto, Colombia Universidad Cooperativa de Colombia Grupo Interdisciplinario de Investigación en Salud y Enfermedad Universidad Cooperativa de Colombia Pasto Colombia; 3 Grupo de Investigación en Genética Humana y Aplicada, Universidad del Cauca, Popayán, Colombia Universidad del Cauca Grupo de Investigación en Genética Humana y Aplicada Universidad del Cauca Popayán Colombia

**Keywords:** Helicobacter pylori, neoplasias gástricas, lesiones precancerosas, gastritis atrófica., Helicobacter pylori, stomach neoplasms, precancerous conditions, gastritis, atrophic

## Abstract

**Introducción.:**

En Asia y Europa, el sistema OLGA ha sido útil como marcador de riesgo de cáncer gástrico. Sin embargo, su utilidad en poblaciones de alto riesgo en Colombia aún se desconoce.

**Objetivo.:**

Establecer si los estadios OLGA se asocian con un mayor riesgo de cáncer y displasia en una población de alto riesgo en Colombia y determinar la capacidad diagnóstica de la escala para evaluar dicho riesgo.

**Materiales y métodos.:**

Se realizó un estudio multicéntrico con pacientes con cáncer gástrico y displasia (casos), y pacientes con atrofia y metaplasia intestinal (controles), provenientes de tres centros de una zona de alto riesgo de cáncer gástrico en Colombia. Se incluyeron 506 pacientes cuyo estudio endoscópico e histopatológico fue realizado mediante el sistema de Sydney y la estadificación de OLGA propuesta por Rugge. El efecto de cada variable de interés sobre la enfermedad (cáncer gástrico y displasia) se evaluó mediante modelos bivariados y multivariados. Un valor de p menor de 0,05 se consideró estadísticamente significativo.

**Resultados.:**

Los estadios elevados del sistema OLGA (III-IV) se asociaron con un mayor riesgo de displasia y cáncer gástrico (OR ajustado = 8,71; IC_95 %_ = 5,09-14,9; p=0,001) con una sensibilidad del 54,9 %, especificidad del 89,3 % y una razón de probabilidad positiva de 5,17.

**Conclusiones.:**

El estadio OLGA es un marcador de riesgo de cáncer gástrico y displasia en la población de estudio. Se recomienda su implementación como estrategia para optimizar el diagnóstico oportuno y el seguimiento de pacientes con mayor riesgo.

## Abstract (en)

**Introduction.:**

The OLGA system has been proved to be useful in Asia and Europe as a risk marker of gastric cancer. However, its usefulness in high-risk populations in Colombia is still unknown.

**Objective.:**

To assess potential associations between the OLGA staging system and an increased risk of gastric cancer and dysplasia in a high-risk Colombian population and to establish diagnostic capacity of the scale to assess the risk.

**Materials and methods.:**

We carried out a multicenter study including patients with cancer and dysplasia (cases) and patients with atrophy and intestinal metaplasia (controls). A total of 506 patients were recruited from three centers in an area with a high risk population in Colombia. The endoscopic and histopathologic studies were evaluated according to the Sydney system and the OLGA staging system proposed by Rugge. The effect of each variable on the disease (gastric cancer and dysplasia) was evaluated using bivariate and multivariate models. Statistical significance was set considering a p value inferior to 0.05.

**Results.:**

Advanced stages of the OLGA system (III-IV) were associated with a higher risk of dysplasia and gastric cancer (adjusted OR = 8.71; CI_95%_ = 5.09-14.9; p=0.001), sensitivity=54.9%, specificity=89.3% and positive likelihood ratio=5.17.

**Conclusions.:**

The OLGA staging system is a risk marker for gastric cancer and dysplasia in the studied population. We recommend its implementation to improve the timely diagnosis and follow-up of patients with the highest cancer risk.

Para el año 2020, el cáncer gástrico fue responsable de 1’089.103 muertes en el mundo y ocupa el quinto lugar como causa de mortalidad por cáncer ^1^. Según Globocan, el 75,3 % de los casos incidentes de cáncer en el mundo ocurren en Asia, 12,5 % en Europa y 6,2 % en Latinoamérica ^1^.

El pronóstico de la enfermedad depende de distintos factores, y el estadio tumoral es el de mayor importancia. Así, cuando la enfermedad se detecta en estadios tempranos, es potencialmente curable, con una supervivencia a cinco años que supera el 90 % de los pacientes ^2^. Sin embargo, cuando la enfermedad se detecta tardíamente, su pronóstico es especialmente sombrío con una supervivencia a cinco años inferior al 10-20 % ^3^. En Asia, los programas de detección temprana han permitido diagnosticar la enfermedad en estadios tempranos con una reducción de la mortalidad del 47 % ^4^.

No obstante, en los países occidentales, especialmente en Latinoamérica, el diagnóstico de cáncer gástrico se hace en etapas avanzadas. Colombia es uno de los países más afectados por la enfermedad y el diagnóstico tardío. Por ejemplo, en el Cauca, considerada una región de alto riesgo de cáncer en Colombia, el 92,4 % de los pacientes con cáncer gástrico tuvo un diagnóstico tardío ^5^.

En este sentido, la prevención y la detección temprana es por el momento la mejor estrategia para mitigar los efectos de la enfermedad. La teoría de la carcinogénesis de múltiples pasos resulta relevante ya que plantea la aparición de lesiones histopatológicas que preceden la aparición del cáncer gástrico ^6^. Según esta teoría, la gastritis crónica, la atrofia, la metaplasia y la displasia son lesiones precursoras de malignidad, es decir, anormalidades histopatológicas previas a la aparición de un cáncer ^7^.

Teniendo en cuenta el carácter progresivo de las lesiones preneoplásicas, la mayoría de los autores coinciden en que el seguimiento de pacientes con dichas lesiones permite un diagnóstico precoz del cáncer y un mejor pronóstico ^8^. No obstante, la tasa de progresión a cáncer es muy baja: 0,1 % para la atrofia y 6 % para la displasia grave ^9^. Además, aún no es posible predecir cuáles pacientes con lesiones preneoplásicas terminarán desarrollando un cáncer. Por ello, se ha propuesto , el sistema OLGA *(Operative Link on Gastritis Assessment)* como estrategia para la estratificación del riesgo. Este sistema evalúa a nivel microscópico el grado de atrofia definido por la disminución de la población glandular o la sustitución del epitelio por células intestinales (metaplasia) ^10^. En este sistema, las muestras tomadas de cinco partes de la mucosa gástrica se evalúan microscópicamente y se clasifican en una escala de 0 al IV según el riesgo progresivo de desarrollar cáncer gástrico, en el que los pacientes con estadios III y IV tienen mayor riesgo de padecer la enfermedad ^10^.

Aunque existe otra herramienta alternativa que evalúa la metaplasia intestinal, llamada OLGIM *(Operative Link on Gastritis Intestinal Metaplasia)*^11^, la capacidad de evaluar cualitativamente la atrofia y la metaplasia, así como la capacidad de evaluar la cascada de carcinogénesis en etapas más tempranas -el modelo de Pelayo plantea que la atrofia antecede la metaplasia- hacen del OLGA una herramienta útil en la estratificación del riesgo. En este sentido, aunque OLGIM es un indicador más reproducible entre los patólogos, algunos autores han demostrado que puede subestimar casos que con OLGA serían de alto riesgo ^12^. Por otra parte, se ha establecido que la valoración de OLGIM plantea varios inconvenientes pues su poder predictivo no estaría asociado *per se* a la presencia de metaplasia, sino al cambio histológico que es sencillamente una de las caras de la atrofia ^13^^,^^14^.

Distintos estudios analíticos de cohortes y de casos y controles han demostrado una asociación consistente entre los estadios elevados del sistema OLGA (III-IV) y el riesgo de desarrollar cáncer gástrico ^15^. Estas investigaciones se han desarrollado en países europeos y asiáticos. No obstante, según el conocimiento de los autores, tal asociación no se ha demostrado en regiones de alto riesgo en países de América como Colombia.

Teniendo en cuenta que los pacientes con algún grado de atrofia (OLGA I a IV) se encuentran en el campo de cancerización (sic) donde ocurren la mayoría de los cánceres gástricos (tanto intestinal como difuso) ^16^^,^^17^, esta investigación está orientada a probar la hipótesis de que las escalas elevadas de OLGA (III y IV) se asocian a un mayor riesgo de cáncer y displasia, y con ello se busca valorar la capacidad diagnóstica de la escala para evaluar dicho riesgo.

## Materiales y métodos

El estudio se llevó a cabo en el departamento del Cauca, donde la tasa global de incidencia de cáncer gástrico es de 22 casos por 100.000 habitantes, por lo que forma parte de los departamentos con incidencia superior a 16 casos por 100.000 habitantes por año y es catalogado como de alto riesgo de acuerdo con la información del Observatorio Nacional del Cáncer de Colombia ^18^.

Se realizó un estudio observacional, analítico, de casos y controles, no pareado. La población de estudio se recolectó entre los años 2008 y 2016, proveniente de los servicios de consulta externa de tres centros de atención en el Cauca: el Hospital Universitario San José de Popayán, la Clínica La Estancia y Endovideo. Los centros mencionados son de tercer nivel de atención, cuentan con servicios de gastroenterología y de patología, y reciben pacientes de los regímenes contributivos y subsidiados del Cauca.

Se incluyeron pacientes mayores de 18 años y que según el diagnóstico histopatológico tuvieran hallazgos de cancerización (atrofia, metaplasia, displasia o adenocarcinoma). Para cada paciente se seleccionó la categoría diagnóstica de mayor gravedad de acuerdo con el modelo de carcinogénesis de Correa ^6^. Se excluyeron pacientes con HIV, cáncer distinto al gástrico, tratamiento previo para *Helicobacter pylori* y pacientes sin atrofia (OLGA 0) dado que por definición no tienen la variable trazadora que se quiere evaluar en este estudio.

Para efectuar los análisis se agruparon los pacientes con diagnóstico histopatológico de displasia y adenocarcinoma como casos, y los pacientes con diagnóstico histopatológico de atrofia y metaplasia como controles.

La endoscopia fue realizada por gastroenterólogos de amplia experiencia. Los pacientes fueron referidos del servicio de consulta externa, contaron con un periodo de ayuno, al menos, de ocho horas y recibieron un anestésico faríngeo de acuerdo con las guías de la Asociación Colombiana de Endoscopia Digestiva ^19^.

Se realizó un muestreo según el protocolo de Sydney; se tomaron, por lo menos, cinco muestras de la mucosa gástrica incluyendo muestras del antro de la curvatura mayor (*incisura angularis*), antro de la curvatura menor, cuerpo de la curvatura mayor y cuerpo de la curvatura menor ^20^^,^^21^. En los pacientes con diagnóstico endoscópico de tumor gástrico se tomaron muestras adicionales del tumor.

Las biopsias se fijaron en formol tamponado al 10% y se colocaron en contenedores separados, debidamente rotulados. Las muestras fueron incluidas en bloques de parafina que luego fueron seccionados en láminas de 4 pm de espesor. Estas se colorearon con hematoxilina y eosina y se usó la tinción de Giemsa para evaluar la presencia de *H. pylori*^22^*.* El diagnóstico histopatológico inicialmente fue efectuado por un primer patólogo experto quien empleó escalas visuales análogas para la categorización de la atrofia en leve, moderada y grave en cada una de las biopsias, según el modelo de Sydney ^20^. El estadio OLGA fue consolidado en los reportes histopatológicos por un segundo patólogo de acuerdo con el sistema propuesto por Rugge ^10^.

Para evitar los sesgos de clasificación, el primer patólogo desconocía el objetivo principal de la investigación. Con el fin de reducir la variabilidad en los casos de displasia de bajo y alto grado, los pacientes se agruparon en una sola categoría (displasia). Por otra parte, para reducir la variabilidad en el análisis de los estadios OLGA, estos se subclasificaron en estadios bajos (I y II) y elevados (III y IV), según lo han propuesto otros autores ^15^.

Una vez comprobada la normalidad de la variable edad mediante la prueba de Kolmogórov-Smirnov, se expresó en los dos grupos de estudio (casos y controles) en términos de media y desviación estándar. Las diferencias de medias se evaluaron mediante la prueba t de Student y las diferencias de proporciones se calcularon a través de la prueba de x^2^. Para estimar el efecto del estadio OLGA en el análisis bivariado se establecieron dos grupos de comparación: OLGA en estadio bajo (I y II como grupo referente) y OLGA en estadio elevado (III y IV). Se calcularon medidas de asociación, mediante razones de probabilidad (*odds ratio*, OR), para el estadio OLGA y las variables de interés como edad, sexo e infección por *H. pylori*.

Para controlar el posible efecto de confusión de la edad se hizo un análisis estratificado entre menores de 50 años y mayores de 50 años. Finalmente, se evaluó el efecto de la escala OLGA sobre el desenlace en un modelo de regresión logística multivariado que incluyó posibles variables de confusión y de interés. Un valor de p menor de 0,05 se consideró estadísticamente significativo. Los análisis estadísticos se realizaron mediante el programa SPSS™, versión 25. Para calcular la validez diagnóstica de OLGA y estimar el riesgo, se calcularon medidas como sensibilidad, especificidad, valores predictivos positivo y negativo, y razones de probabilidad positiva y negativa.

Los participantes del estudio aceptaron voluntariamente su participación y firmaron un consentimiento informado. La investigación fue aprobada por el Comité de Ética para la Investigación Científica de la Universidad del Cauca.

## Resultados

Durante el periodo de estudio se recolectaron muestras de 1.243 pacientes, de los cuales, 506 cumplieron con los criterios de inclusión. De ellos, 91 correspondían a pacientes con displasia y cáncer (casos) y 415 a pacientes con atrofia y metaplasia intestinal (controles) con una relación de 4,5 controles por cada caso. El rango de edad de los participantes osciló entre los 20 y los 87 años.

La distribución de los participantes del estudio de acuerdo con el diagnóstico histopatológico fue: 301 (59 %) con metaplasia intestinal, 114 (23 %) con gastritis crónica atrófica, 51 (10 %) con displasia y 40 (8 %) con adenocarcinoma gástrico.

En el cuadro 1 se muestra la distribución de las variables de interés en relación con los diagnósticos histopatológicos. La mayoría de los pacientes con displasia y cáncer correspondió a individuos mayores de 50 años y de sexo masculino.

**Cuadro 1:** Distribución de las variables de estudio de acuerdo con el diagnóstico histopatológico

		Gastritis atrófica n (%)	Metaplasia intestinal n (%)	Displasia n (%)	Adenocarcinoma gástrico n (%)
Edad (años)	18 a 50	42 (36,8)	118 (39,2)	10 (19,6)	2 (5)
	> 51	72 (63,2)	183 (60,8)	41 (80,4)	38 (95)
Sexo	Femenino	78 (68,4)	192 (63,8)	27 (52,9)	16 (40)
	Masculino	36 (31,6)	109 (36,2)	24 (47,1)	24 (60)
*Helicobacter pylori*	Negativo	38 (33,3)	59 (19,6)	11 (21,6)	9 (22,5)
	Positivo	76 (66,7)	242 (80,4)	40 (78,4)	31 (77,5)
Estadio OLGA	I	85 (74,6)	117 (38,9)	0 (0)	7 (17,5)
	II	29 (25,4)	140 (46,5)	15 (29,4)	19 (47,5)
	III	0 (0)	33 (11)	25 (49)	10 (25)
	IV	0 (0)	11 (3,7)	11 (21,6)	4 (10)

Después de comprobar la normalidad para la variable edad en el grupo de casos y controles con la prueba de Kolmogórov-Smirnov (p=0,078 para los dos grupos), se procedió a realizar una comparación de medias con la prueba t de Student. La media para el grupo control fue de 55 años mientras que en el grupo de pacientes con displasia y cáncer fue de 65 años. La prueba t de Student mostró diferencias significativas entre las medias de los dos grupos (p=0,001)

La edad categorizada, el sexo y los estadios OLGA también mostraron diferencias significativas entre los grupos. Por su parte, la presencia de infección por *H. pylori* no mostró diferencias entre los casos y los controles (cuadro 2).

**Cuadro 2:** Diferencias de proporciones entre las variables de interés

		Controles (gastritis atrófica y metaplasia) n (%)	Casos (displasia y cáncer gástrico) n (%)	p
Edad (años)	18 a 50	160 (38,6)	12 (13,2)	0,001^a^
	> 51	255 (61,4)	79 (86,8)	
Sexo	Femenino	270 (65,1)	43 (47,3)	0,002^a^
	Masculino	145 (34,9)	48 (52,7)	
*Helicobacter pylori*	Negativo	97 (23,4)	20 (22)	0,775^a^
	Positivo	318 (76,6)	71 (78)	
Estadio OLGA	I	202 (48,7)	7 (7,7)	0,001^a^
	II	169 (40,7)	34 (37,4)	
	III	33 (8)	35 (38,5)	
	IV	11 (2,7)	15 (16,5)	

^a^ Se realizó una prueba de *x* ^2^ para comparar las proporciones. Un valor de p<0,005 se consideró estadísticamente significativo.

Para estimar el efecto de las variables de interés sobre el desenlace se procedió a calcular las razones de probabilidad tomando como categorías de referencia la edad menor de 50 años, el sexo femenino y la ausencia de infección por *H. pylori.* Para probar la hipótesis principal del estudio se compararon los estadios altos de OLGA (III-IV) y se contrastaron con los estadios bajos (I-II).

Dado que la edad mayor de 50 años fue más prevalente en el grupo de casos, se procedió a realizar un análisis estratificado en dos categorías: pacientes menores de 50 años y mayores de 50 años. El grupo de participantes menores de 50 años incluyó 172 pacientes, 160 pertenecientes al grupo control y 12 al grupo de casos. En esta categoría de edad, la razón de probabilidad de la asociación entre los estadios OLGA elevados con las variables desenlace (displasia y cáncer) fue de 13,45 (IC_95 %_ = 3,74-49,04; p<0,001).

Para la segunda categoría de edad (pacientes mayores de 50 años) se tuvieron 334 pacientes: 255 controles y 79 casos. El cálculo de la razón de probabilidad de la asociación entre los estadios elevados de OLGA y la variable desenlace fue de 8,45 (IC_95 %_ = 4,75-15,03; p<0,001).

Finalmente, dado que en los análisis bivariados se encontraron asociaciones significativas de las variables de interés (edad, sexo y estadios OLGA) con el desarrollo de displasia y cáncer, estas variables se incluyeron en un modelo de regresión logística multivariado con el fin de evaluar posibles factores de confusión. A pesar de que la infección por *H. pylori* no mostró una asociación significativa en el modelo bivariado, también se incluyó en el análisis multivariado dada la reconocida relación descrita en la literatura con el desarrollo de displasia y cáncer (cuadro 3).

**Cuadro 3:** Medidas de asociación crudas y ajustadas en un modelo de regresión logística multivariado

		OR crudo	^IC^ **95 %**	p	OR ajustado	IC_95 %_	p
Edad (años)	18 a 50	1	1	1	1	1	1
	> 51	4,13	(2,18-7,82)	0,001	3,14^a^	(1,58-6,23)	0,001
Sexo	Femenino	1	1	1	1	1	1
	Masculino	2,08	(1,31-3,28)	0,002	1,50^b^	(0,89-2,53)	0,125
*Helicobacter pylori*	Negativo	1	1	1	1	1	1
	Positivo	1,08	(0,62-1,87)	0,775	0,92^c^	(0,49-1,73)	0,808
Estadio OLGA	I-II	1	1	1	1	1	1
	III-IV	10,28	(6,12-17,26)	0,001	8,71^d^	(5,09-14,9)	0,001

OR: razón de probabilidad; IC: intervalo de confianza

^a^ Modelo de regresión ajustado por sexo, infección por *Helicobacter pylori* y estadio OLGA

^b^ Modelo de regresión ajustado por edad, infección por *Helicobacter pylori* y estadio OLGA

^c^ Modelo ajustado por edad, sexo y estadio OLGA

^d^ Modelo de regresión ajustado por edad, sexo e infección por *Helicobacter pylori*. Un valor de p<0,05 se consideró estadísticamente significativo.

La capacidad diagnóstica para evaluar el riesgo de displasia y cáncer en pacientes con estadios OLGA elevados se analizó de acuerdo con la información compilada en el cuadro 4.

**Cuadro 4:** Distribución de los pacientes de acuerdo con la clasificación diagnóstica OLGA

OLGA	Casos (n)	Controles (n)	Total (n)
Elevado	50	44	94
Bajo	41	371	412
	91	415	506

Los estadios OLGA elevados incluyen las categorías III y IV, mientras que los bajos incluyen las categorías I y II.

En el cuadro 5 se resumen las estadísticas de capacidad diagnóstica. Se observó que la escala OLGA tiene una especificidad cercana al 90 % y una importante capacidad discriminatoria según las razones de probabilidad calculadas.

**Cuadro 5:** Resultados de validez de los estadios elevados OLGA para estimar el riesgo de displasia y cáncer gástrico

	Resultado	**IC**_95%_
Sensibilidad (%)	54,9	(50,6-59,2)
Especificidad (%)	89,3	(89,2-89,4)
Valor predictivo positivo (%)	53	
Valor predictivo negativo (%)	90	
Razón de probabilidad positiva	5,17	
Razón de probabilidad negativa	0,50	

## Discusión

En este estudio se documenta una asociación entre la edad y el riesgo de displasia y cáncer, también resaltada por otros autores ^23^^,^^24^. La relación de los estadios OLGA se controló en el estudio mediante un análisis estratificado por edad y en un modelo de regresión logística. En ambas exploraciones se estableció que la edad se comporta como una variable predictora independiente del estadio OLGA. Esta conclusión es similar a la planteada por Mansour y colaboradores quienes documentaron casos elevados de OLGA incluso en pacientes menores de 40 años, quienes a pesar de la edad, debían continuar en seguimiento endoscópico ^25^. Estos hallazgos permiten plantear que la atrofia y la presencia de cancer de la mucosa gástrica son factores independientes de la edad y que representan quizá una adaptación pobre frente a otros factores carcinogénicos como el estrés oxidativo, la dieta, la infección por *H. pylori* o la respuesta inflamatoria crónica ^26^.

El estudio histológico en esta investigación mostró prevalencias de infección bacteriana superiores al 70 %, similares a las reportadas en zonas de alto riesgo de Colombia por otros autores ^27^. Algunos investigadores han evaluado el papel de la infección bacteriana y su interacción con la escala OLGA y han demostrado que la infección bacteriana aumenta el riesgo de cáncer tanto en el modelo bivariado como en el modelo ajustado por los estadios OLGA ^28^. Estos hallazgos difieren de los resultados de la presente investigación donde no se pudo establecer tal asociación ni en las estimaciones crudas, ni en la regresión logística.

Como explicación a este fenómeno, cabe resaltar que en publicaciones previas sobre la misma población de estudio se ha establecido que el mayor riesgo de cáncer no obedece únicamente a la presencia de *H. pylori*, sino a su variabilidad genética. En particular, los genotipos *vacA s1* y *m1* son los responsables de un mayor riesgo de desarrollar cáncer gástrico y displasia ^29^. Teniendo en cuenta la elevada prevalencia de la infección bacteriana en la población de estudio, en futuras investigaciones resultaría interesante explorar el efecto interactivo de las variantes genéticas de *H. pylori* con la escala OLGA*.*

Los resultados de esta investigación muestran una asociación entre los estadios elevados de OLGA con displasia y cáncer tanto en el análisis bivariado como en el modelo multivariado. En el modelo multivariado controlado por edad, sexo e infección por *H. pylori*, se demostró que los pacientes con displasia y cáncer tienen un riesgo 8,71 veces mayor de estar en estadios elevados de OLGA comparados con los controles. Estos hallazgos son consistentes con los publicados en el año 2018 por Yue y colaboradores en el que realizaron un metaanálisis con 2.700 pacientes, en el que se incluyeron estudios de casos y controles y estudios de cohortes, para evaluar la asociación entre los estadios OLGA y el riesgo de cáncer. En ese metaanálisis, los estudios de casos y controles (OR=2,64; IC_95 %_ = 1,84-3,79; p<0,00001) y los de cohortes [riesgo relativo (RR)=27,7 IC_95 %_ = 3,75-204,8; p<0,001] demostraron que la clasificación en los estadios III y IV de OLGA aumenta el riesgo de padecer cáncer ^15^.

En las Américas, hay pocas investigaciones de tipo descriptivo o analítico que evalúan la prevalencia de los estadios OLGA o que exploran la capacidad de ese sistema para detectar atrofia mediante un muestreo múltiple ^30^^-^^32^. Sin embargo, según el conocimiento de los autores, no se han desarrollado investigaciones con el fin de establecer si los estadios OLGA en poblaciones de alto riesgo de las Américas se asocian con un riesgo diferencial de cáncer de estómago. Por lo tanto, la presente investigación es la primera que explora desde un enfoque analítico, la asociación de la escala OLGA con el riesgo de cáncer gástrico y displasia en una población colombiana. Este sería también el primer estudio de este estilo publicado en una población de alto riesgo de las Américas, en el cual se estima la capacidad diagnóstica del riesgo.

En este estudio, las medidas de evaluación del OLGA como una prueba para diagnosticar el riesgo de displasia o cáncer mostraron una especificidad y un valor predictivo negativo cercanos al 90 %. Estos hallazgos sugieren en principio que, ante la ausencia de un estadio elevado de OLGA, el riesgo de displasia y cáncer sería bajo. Por lo tanto, la recomendación que han hecho varios autores y guías de espaciar los controles endoscópicos e histopatológicos en pacientes con estadios bajos de OLGA estaría acorde con los resultados del presente reporte ^33^^-^^35^.

Por otra parte, en esta investigación se muestra que la sensibilidad y el valor predictivo positivo de la escala OLGA para detectar riesgo de malignidad fueron del 55 y 53 %, respectivamente. Estos porcentajes resaltan quizá el carácter multifactorial del cáncer, cuyo riesgo es difícil de calcular mediante una sola estimación. Como lo sugieren Saka y colaboradores, la sensibilidad de este marcador histológico puede mejorarse mediante la combinación de sistemas de clasificación que involucren otras variables como los hallazgos endoscópicos ^36^. Actualmente, el equipo de investigación de los autores trabaja en el desarrollo de una escala que, además de validar OLGA, evalúe otras variables histopatológicas (tipo OLGIM), el patrón de respuesta inflamatoria, los factores dietéticos y la variabilidad genética de *H. pylori* para lograr un índice predictivo más sensible.

Los resultados de la regresión logística, así como las razones de probabilidad positiva y negativa calculadas en este estudio indican un poder discriminatorio significativo. Las razones de probabilidad resaltan, por ejemplo, que por cada paciente de bajo riesgo con un resultado elevado de la escala OLGA, se presentan cinco pacientes de alto riesgo con resultados elevados en la misma escala; mientras que, por cada paciente de alto riesgo con estadios bajos de OLGA, se presentan dos pacientes de bajo riesgo con estadios bajos en OLGA. Este poder de discriminación plantea la escala OLGA como una herramienta útil en un país como Colombia, donde cerca del 90 % de los casos de cáncer se diagnostican en estadios avanzados y donde no existe una política de salud pública para mitigar la enfermedad ^37^. Dado que el factor pronóstico más importante en la evolución del cáncer es precisamente el diagnóstico oportuno, la estadificación OLGA es una estrategia prometedora ya que permitiría el seguimiento periódico y estratégico de los pacientes con mayor riesgo, y optimizaría la tasa de diagnósticos precoces de la enfermedad.

En resumen, se propone incluir la escala OLGA de manera rutinaria en los estudios histopatológicos para la estratificación del riesgo de cáncer gástrico, en particular, en zonas de alta incidencia de cáncer en Colombia. Al igual que otros autores, este estudio considera que la escala brinda una herramienta clínica sólida con sustento biológico para racionalizar el seguimiento de los pacientes ^17^. En un trabajo previo de Martínez y colaboradores, se estableció que el muestreo amplio de la mucosa gástrica con cinco biopsias permite detectar más casos de atrofia que muestreos subóptimos ^30^. Estos resultados aunados a los del presente trabajo permiten concluir que el muestreo amplio de la mucosa gástrica (incluída la *incisura angularis*), no solo permite diagnosticar pacientes que se encuentran en el campo de desarrollo del cancer, sino también estratificar su riesgo individual. Por lo tanto, se sugiere realizar un muestreo múltiple de la mucosa gástrica con la toma de cinco biopsias según el protocolo de Sydney, enviar las muestras en contenedores separados, debidamente rotulados, y anexar en los reportes histopatológicos el estadio OLGA. Esta estrategia, basada en evidencia de población colombiana, apoya las recomendaciones de la guía de práctica clínica para la prevención primaria y detección del cáncer gástrico en poblaciones de alto riesgo ^38^ y podría establecerse como una política de salud pública para la detección oportuna de la enfermedad. Para lograr su implementación, se requiere de un trabajo articulado de endoscopistas, gastroenterólogos y patólogos que permita mejorar el diagnóstico oportuno del cáncer gástrico y optimizar los recursos y esfuerzos médicos en la población de mayor riesgo.

Una de las limitaciones de este estudio deriva del tipo de diseño, toda vez que la noción de causalidad se infiere de manera indirecta en los estudios de casos y controles. Por ello, para futuros estudios se recomienda el desarrollo de estudios de cohortes en los que la incidencia de la enfermedad y la noción del riesgo se puedan atribuir de forma directa. A pesar de las limitaciones del diseño, se trabajaron distintos aspectos como los criterios de calidad considerados en la publicación de Yue y que incluyen tamaño muestral mayor de 100 participantes, definición de caso y control, comparabilidad de los grupos, comprobación de la exposición y paridad de los métodos diagnósticos para evaluar los casos y controles.
